# Health Tract No. 350

**Published:** 1868-11

**Authors:** 


### House Building.

There is not a man in the universe who has ever built a comfortable, safe, healthful house. There is not an architect in existence who has sense enough to construct a dwelling fit for a man to live in, or the man can't be found who is liberal enough to furnish the money to do it. Who has ever seen a church that did not leak? who has ever heard of a church, which for want of ventilation does not sicken or put to sleep, half of its regular attendants? who ever saw a church which the devil did not have a hand in building so as to prevent three-fourths of the congregation from hearing what the minister says, without a painful effort. The late Dr. James W. Alexander expressed to us the same idea shortly before he died in reference to Spurgeon's preaching, an important element of which he thought was the distinctness and easiness with which he was heard, but in his pure and courtly manner he put it in this form, "I am afraid the adversary has too much to do in the building of our American churches." What millions of money are lost every year by defective flues! and this last because in fifty years' experience our architects have not learned that wood will become tinder if kept in a heated condition for a whole winter, when literally a spark will set it on fire; and may almost be said to be spontaneously combustible, and that too when the certain remedy is *not* to have a piece of wood within a foot of the flue, or to have the flue encased with several inches' thickness of some non-conductor of heat, thus saving an immense amount of heat. Who has not been annoyed many a time at being kept awake by the shutting of a window sash, or the flapping of an outer shutter of a stormy night; and being obliged to risk health, neck and life by getting up and poking the head out in the storm to fasten the shutter. Can nobody invent a shutter which can be opened and closed without hoisting the sash? Has no living man genius enough to devise a sash which may be hoisted easily and at the same time never rattle? And as to ventilation of our sitting and sleeping rooms. Listen to our dicta, O ye numskulls! There is no healthfulness in sleeping in a room cooler than fifty degrees; in cold countries the most equable, economical and healthful heat in the world is made by a close stove, or an old fashioned sheet iron stove of fifty years ago, all that you have to do is to regulate the heat and moisture of the room and not only drive the bad air out, but invite pure air in. Edson's Hydrometer will tell you the actual amount of moisture in a room and how to have more or less, sixty grains of water to each cubic foot of air being the healthful point. If you will heat a room by flues, let them be immediately under the ceiling with holes in a furred floor, then the new hot air drives the old warm air through the floor, which passing under it keeps it always warm, and you breathe constantly a pure, warm air.

